# Medical Department of Niagara University

**Published:** 1896-05

**Authors:** 


### MEDICAL DEPARTMENT OF NIAGARA UNIVERSITY.

THIS flourishing medical school closes its thirteenth year on on Tuesday, May 12, 1896. The board of medical examiners of the University will meet at the college building on Ellicott street, at 10 A. M. that day, to examine such candidates for graduation as the medical faculty may present to it. Those which both the faculty and examiners recommend to the trustees as worthy will receive the degree of Doctor of Medicine in the evening, when the commencement exercises will take place at the Star theater. The degrees will be conferred by the chancellor of the University according to the ancient rite of "hooding." Dr. Herman Mynter, of the faculty, will deliver the valedictory to the class, and the Right Rev. Willard F. Mallalieu, bishop of the Methodist Episcopal Church, of Buffalo, will give the charge to the graduates.

During the day, beginning at 10.30 A. M., the Alumni Association will meet, under the presidency of Dr. Joseph J. Kane, of Buffalo. The program will embrace an address of welcome by a member of the faculty, the annual address of the president, and papers by Drs. Sydney A. Dunham, Charles E. Congdon, David L. Redmond and Lawrence G. Hanley, of Buffalo, and Dr. J. S. Peterson, of New York City, all alumni of the university.

The alumni banquet will be held at the Genesee hotel immediately on the conclusion of the commencement exercises.

The medical profession is cordially invited to all these exercises, to which all friends of the college will be given a hearty welcome.

